# The Impact of a Multifaceted Pharmacist-Led Antimicrobial Stewardship Program on Antibiotic Use: Evidence From a Quasi-Experimental Study in the Department of Vascular and Interventional Radiology in a Chinese Tertiary Hospital

**DOI:** 10.3389/fphar.2022.832078

**Published:** 2022-02-28

**Authors:** Jinhui Xu, Jian Huang, YanXia Yu, Dayong Zhou, Ying Wang, Sudong Xue, Erning Shang, Jiantong Sun, Xinyuan Ding, Lu Shi, Lufen Duan, Lian Tang, Qin Zhou, Xin Li

**Affiliations:** ^1^ Department of Pharmacy, The Affiliated Suzhou Hospital of Nanjing Medical University, Suzhou, China; ^2^ Department of Vascular and Interventional Radiology, The Affiliated Suzhou Hospital of Nanjing Medical University, Suzhou, China; ^3^ Department of Education and Training, The First Affiliated Hospital of SooChow University, SuZhou, China; ^4^ Department of Clinical Pharmacy, School of Pharmacy, Nanjing Medical University, Nanjing, China; ^5^ Center for Global Health, School of Public Health, Nanjing Medical University, Nanjing, China

**Keywords:** antibiotics use, antimicrobial stewardship, pharmacist, difference-in-differences, vascular and interventional radiology

## Abstract

**Objective:** The objective of the study was to assess the impact of multifaceted clinical pharmacist-led antimicrobial stewardship (AMS) program on the rational use of antibiotics for patients who receive vascular and interventional radiology therapies.

**Methods:** A quasi-experimental retrospective intervention design with a comparison group was applied to the practice of antibiotic use in the department of vascular and interventional radiology in a Chinese tertiary hospital. We used difference-in-differences (DID) analysis to compare outcomes before and after the AMS intervention between the intervention group and control group, to determine whether intervention would lead to changes in irrationality of antibiotic prescribing, antibiotic utilization, cost of antibiotics, and length of hospital stay.

**Results:** The DID results showed that the intervention group was associated with a reduction in the average consumption of antibiotics (*p* = 0.017) and cost of antibiotics (*p* = 0.006) and cost per defined daily dose (DDD) (*p* = 0.000). There were no significant differences in the mean change of total costs and length of stay between the two groups (*p* > 0.05). The average inappropriate score of perioperative antimicrobial prophylaxis in the intervention group declined by 0.23, while it decreased by 0.02 in the control group [0.21 (95% CI, −0.271 to −0.143); *p* = 0.000]. The average inappropriate score of non-surgical antimicrobial prophylaxis in the intervention group declined by 0.14, while it increased by 0.02 in the control group [0.16 (95% CI, −0.288 to −0.035); *p* = 0.010]. The average inappropriate score of the therapeutic use of antibiotics in the intervention group declined by 0.07, while it decreased by 0.01 in the control group [0.06 (95% CI, −0.115 to −0.022); *p* = 0.003].

**Conclusions:** This study provides evidence that implementation of AMS interventions was associated with a marked reduction of antibiotic use, cost of antibiotics, and irrationality of antibiotic prescribing in China.

## Introduction

By making lethal infections readily treatable, antibiotics have led the development of modern medicine. Prompt initiation of appropriate antibiotic therapy to treat infectious diseases reduces morbidity and save lives. Moreover, antibiotics also enables multiple advances in the area of medical technology, such as organ transplants and cancer chemotherapy (Beyar-Katz et al., 2017; Versporten et al., 2018). However, about 30%–50% of all antibiotics prescribed in hospitals are either unnecessary or suboptimal worldwide (Denny et al., 2019; Rowe and Linder, 2019). In recent years, antimicrobial resistance (AMR) has been recognized as a growing public health problem of huge concern to countries around the world (Huemer et al., 2020). Inappropriate antibiotic use contributes greatly to the development of AMR (Bohmer et al., 2021), leading to ineffective therapy, increase in adverse reactions, and escalation in health care costs. In order to optimize antibiotic use in medical care, many different antimicrobial stewardship (AMS) initiatives have been conducted in some developed countries recently. Several studies have shown that AMS can help physicians increase infection cure rates, improve treatment outcomes, and reduce antibiotic resistance by improving antibiotic prescribing (Dellit et al., 2007; Rice, 2018).

According to the Centers for Disease Control and Prevention (CDC) of the United States, core elements of hospital AMS programs are as follows: 1) Hospital leadership commitment. AMS programs should be supported from the senior leadership of the hospital, especially the director of pharmacy. 2) Pharmacy expertise. AMS programs should appoint a physician or pharmacist as the coleader to improve antibiotic use. 3) Accountability. As a leader or coleaders, a physician or pharmacist should be responsible for program management. 4) Interventions. The prospective audit and feedback or preauthorization should be implemented to improve antibiotic use. 5) Education. AMS should educate clinicians, pharmacists, nurses, and patients about adverse reactions caused by antibiotics, AMR, and optimal prescribing (CDC, 2019).

A recent study found that 25% (21%–29%) of antibiotic prescriptions in the United States ambulatory care clinics were inappropriate in 2015 (Ray et al., 2019). The rates of inappropriate prescriptions were highest for otitis externa (67.3%) and upper respiratory tract infection (38.7%) in the United Kingdom general practice between January 2010 and June 2015 (Nowakowska et al., 2019). Due to its huge antibiotic consumption (Zhao et al., 2018), strong economic incentives (Lin et al., 2020), and limited knowledge about antibiotics (Liu et al., 2019) for overprescribing, inappropriate antibiotic use in China is common. The proportion of inappropriate antibiotic prescriptions among outpatients and inpatients was 60.6% and 75.4% in primary health care settings in China from 2009 through 2011, respectively (Wang et al., 2014). A recent survey conducted on the use of antibiotics in 52 hospitals of China reported that the proportion of antibiotic prescription in surgical inpatients in 2016 was 69.03% (Li et al., 2019), which did not meet the goal of not more than 60% set by the National Health Commission of the People’s Republic of China (National Health Commission of China, 2011). This report also showed that the percentage of antibiotic use in inpatients for acute upper respiratory infections and fever or cough symptoms all exceeded 55%, which was unnecessary for these infectious diseases mostly caused by viruses (WHO, 2011).

Thus, to promote rational use of antibiotics, it is necessary to implement AMS programs in hospitals. In 2011, AMS policy was formally implemented by the National Health Authority in medical institutions nationwide. Successful implementation of AMS requires a multidisciplinary organizational approach, with pharmacists as the core members that deliver the interventions to optimize antimicrobial use.

Several studies provided evidence that pharmacist-led interventions can improve adherence to guidelines and reduced the duration of antimicrobial therapy with substantial cost savings for patients (Mahmoudi et al., 2019; Monmaturapoj et al., 2021). Furthermore, implementation of pharmacy-based interventions in AMS programs can reduce antibiotic consumption, length of hospital day, and 30-day mortality (Xin et al., 2019; Du et al., 2020).

Previous studies assessed the effectiveness of AMS intervention on surgical antibiotic prophylaxis (Zhang et al., 2014; Abubakar et al., 2019), upper or lower respiratory tract infection (Shen et al., 2011; Foolad et al., 2018), nonspecified indications (Li et al., 2017; May et al., 2021) in those priority departments, such as the department of urology, department of obstetrics and gynecology, department of respiratory medicine, intensive care unit, and emergency department, given high antimicrobial prescribing for prophylaxis or treatment and concerns with inappropriate antibiotic use. However, the previous studies pay little attention to some emerging departments with innovative technology; for instance, as the number and breadth of vascular and interventional radiology procedures grow, it is critical to promote rational use of antibiotics in the department of vascular and interventional radiology to avoid serious infectious complications. So far, only one study on the use of antibiotics for perioperative interventional surgery was retrieved. Yang et al. (2017) conducted a clinical pharmacist-led guidance team intervention for the rationality of prophylactic antibiotic usage for eight interventional procedures in a Chinese tertiary teaching hospital. However, it did not focus on a specific department, and the intervention included mainly with prescription reviews and administrative approach. Moreover, it was only limited to perioperative antimicrobial prophylaxis, rather than comprehensive intervention involving antibiotic prophylaxis and antibiotic therapy in surgical wards. The research design of the study was that of a before-and-after intervention trial to evaluate the effectiveness.

To date, there is no evidence to demonstrate the effectiveness of pharmacist-led AMS interventions on the rational use of antibiotics in the department of vascular and interventional radiology in a Chinese tertiary hospital. Therefore, the aim of this study was to improve the rational antibiotics use in the department of vascular and interventional radiology, and assess the value of pharmacist-led AMS programs during the study period. For this purpose, we used a quasi-experimental study design with a difference-in-differences (DID) analysis comparing outcomes before and after the AMS intervention between intervention group and control group, to determine whether intervention would lead to changes in the following outcomes: irrationality of antibiotic prescribing, antibiotic utilization, cost of antibiotics, and length of hospital stay.

## Materials and Methods

### Study Setting

This was a quasi-experimental retrospective study, with a comparison group and pre- and post-intervention measures in two independent vascular and interventional radiology wards of a tertiary teaching hospital. The two independent wards were randomized into control and intervention groups. In the intervention group, a clinical pharmacist-led AMS was implemented. While in the control group, treatment strategies were performed by the physicians and nurses without pharmacist involvement.

To ensure comparability of the statistical data, inclusion and exclusion criteria were established. Diseases involving the circulatory system (such as superficial thrombophlebitis of the lower extremities, aortic aneurysm or dissection, etc.), digestive system (such as biliary obstruction, cirrhosis with esophageal and gastric variceal bleeding, etc.), respiratory system (bronchiectasis, pneumonia, etc.), genitourinary system (such as urinary tract infection, kidney failure, etc.), tumor (liver malignant tumor), injury, poisoning, certain other consequences of external causes (such as complications of procedures, injury of kidney, etc.), the blood and blood-forming organs, and certain disorders involving the immune mechanism, skin, and subcutaneous tissue, and congenital malformation (such as cellulitis, polycystic kidney, hypersplenism, etc.) were enrolled. Patients were excluded if they were transferred from other medical departments, were transferred to other medical departments for further treatment, and ages were younger than 18 years or data were missing. Accordingly, in our retrospective study, phases were divided into two stages as follows: pre-intervention period (March 2018 to October 2018) and post-intervention period (March 2019 to October 2019).

### Ethical Consideration

The retrospective study complied with the Declaration of Helsinki, and ethical approval was obtained from the Ethical Committee of Nanjing Medical University (grant number: ethical review 2020103).

### Pharmacist-Led Antimicrobial Stewardship Interventions

Based on the core elements of AMS (CDC, 2019), the multifaceted interventions included: 1) Establishment of criteria: provided practitioners with a standardized approach to the safe and effective use of antibiotic drugs based on global and national guidelines. 2) Daily ward round: participated in physicians’ ward rounds at working days only and made recommendations on the antimicrobial prescribing. 3) Regular review of medical orders: communicated with the physicians in cases of inappropriate drug use and made suggestions to determine the optimal use of antibiotics. 4) Routine education and training: trained the medical staff on rational use of antibiotics and correct microbiology specimen submission. 5) Consultations and case discussions: conducted regular multidisciplinary consultations and case seminars for difficult cases to discuss diagnosis and modify the regimen.

### Data Collection and Outcomes

The primary outcome was the score of inappropriate antimicrobial use between pre-intervention and post-intervention period, which was evaluated by the multidisciplinary team. The team consisted of an infectious disease specialist, a ward physician, and a clinical pharmacist, who were blinded to the patients’ allocation status. The appropriateness of antimicrobial use was assessed by the three specialists, respectively. If the score was the same, the result would be adopted, when there was any disagreement, final decision would be made after a discussion.

Secondary outcomes included antimicrobial consumption, antimicrobial cost, the average cost per defined daily dose (DDD), the total cost of hospitalization, and length of hospital stay per patient during the periods. Antimicrobial consumption was defined as the defined daily doses (DDDs) of antibiotics used per patient that was applied in previous studies of antibiotic utilization (Yang et al., 2017; Abubakar et al., 2019). The average cost per DDD was calculated by dividing the antimicrobial cost by DDDs of antibiotics used per patient.

Clinical data including patients’ baseline characteristics, antibiotic usage, cost, length of hospital stay, and clinical outcome were extracted from the hospital information system (HIS). The HIS recorded the clinical outcome of the discharged patients, which were classified as cure, improvement, failure, and death. Interventions identified by the pharmacist-led AMS program and the accepted recommendations were collected within the electronic medical record. The use of antibiotics was stratified into three indication categories: perioperative antimicrobial prophylaxis, non-surgical antimicrobial prophylaxis, and therapeutic use of antibiotics. The specialists evaluated the phenomena of inappropriate drug use using a scoring system that consisted of different items regarding the three categories. Each item was assigned one point if an inappropriate phenomenon was identified. At the same time, the score was multiplied by the weight of each irrational medication indicator, which was developed by 30 experts’ consultation using the Saaty 1–9 scaling method (Saaty, 1990)*.*

### Statistical Analysis

In this study, the variations in an index of the two groups before and after intervention are calculated to reflect the net effect of clinical pharmacist-led AMS strategy. A DID methodology (Wooldrige, 2002; Dimick and Ryan, 2014) was used to compare pre- and post-intervention changes between the AMS intervention group and the control group. The differences lie in the cluster-level summaries of the two groups. Comparisons were carried out using a mixed effect linear model with repeated measures using group, time, and group × time as fixed effects, while group is a random effect nested within the study group. The descriptive statistics of cost, DDDs, score, and days are presented as means (by time and by group), and mean changes by group. The DID methodology allows the researchers to control confounding influences of independent variables, as well as imbalance between groups in dependent variables, which are due to chances of imperfect randomization. For each group, the incremental effect of the pharmacist-led AMS intervention was computed as the differences between the pre- and post-implementation of intervention, and then the incremental effects were compared between the intervention and control groups to estimate the net effect of the intervention. The model that was used for this DID regression is written as:

$$Y_{it}=\alpha_{0}+\beta \times time_{it}\times group_{it}+\gamma \times X_{it}+\varepsilon_{it}$$

where i indicates the vascular and interventional radiology ward, and t indicates the time. Y represents the main outcomes, either score of inappropriate antimicrobial use, antimicrobial consumption, cost, or length of hospital stay; time_it_ is the time dummy variable, where it was measured at the pre-intervention period as 0 and post-intervention period as 1; group_it_ is the treatment dummy variable, where the intervention group is 1, while the control group is 0; time_it_ × group_it_ is an interaction term between the time dummy variable and treatment dummy variable, and its coefficient β captured the average effects of the pharmacist-led AMS strategy on the outcome indicators. X_it_ is a series of covariates, including age, sex, clinical outcome, and diagnosis. ε_it_ is a residual error. Each outcome was regressed on the main effects for intervention (1 = intervention group; 0 = control group) and time period (1 = post-intervention, 0 = pre-intervention). In this procedure, the DID model was used to predict the expected value at baseline and follow-up in the intervention and control groups with all other fixed effects set to their mean values.

Continuous variables that were normally distributed were presented as the mean ± standard deviation, and group differences were compared using an independent t-test. Non-normally distributed continuous variables were described in the median and 25th and 75th interquartile range (Q25, Q75) and were assessed using Mann–Whitney U-test. Categorical variables were presented as frequencies and percentages and assessed using the Chi-squared test or Fisher’s exact test. All statistical analyses were conducted using SPSS Statistics 23.0 (IBM Corp., Armonk, NY, USA), and differences with *p* < 0.05 were considered statistically significant.

## Results

### Patient Characteristics

A total of 1,026 patients were enrolled in the study, 514 for the control group and 512 for the intervention group. There were no significant differences in age, gender, number of diagnoses, and clinical outcome of the admitted patients between the two groups (Table 1).

**TABLE 1:** Comparison of the characteristics of patients in the control group and intervention group.

Characteristic	Control group (*n* = 514)	Intervention group (*n* = 512)	*p*-Value
Age, year	67 (57,75)	67 (58,75)	0.607^a^
Male, n (%)	332 (64.59%)	323 (63.08%)	0.616
Diagnosis, n (%)			
Circulatory system	181 (35.21%)	161 (31.44%)	0.200
Digestive system	139 (27.04%)	161 (31.44%)	0.121
Respiratory system	81 (15.76%)	69 (13.48%)	0.301
Genitourinary system	60 (11.67%)	66 (12.89%)	0.552
Tumor	31 (6.03%)	29 (5.66%)	0.802
Injury, poisoning, and certain other consequences of external causes	20 (3.89%)	23 (4.49%)	0.631
Others^b^	14 (2.72%)	19 (3.71%)	0.370
Multiple diagnosis	12 (2.33%)	16 (3.12%)	0.437
Clinical outcome			0.993
Cure	504	502	
Improvement	10	10	
Failure	0	0	
Death	0	0	

Note. Values are presented as median (interquartile range) or n (%). All *p*-values are calculated using Chi-square unless otherwise noted.

a Mann–Whitney U-test.

b Others: the blood and blood-forming organs and certain disorders involving the immune mechanism; skin and subcutaneous tissue; and congenital malformation.

### The Effects of Antimicrobial Stewardship Program on the use of Antibacterial Drugs

**FIGURE 1:**
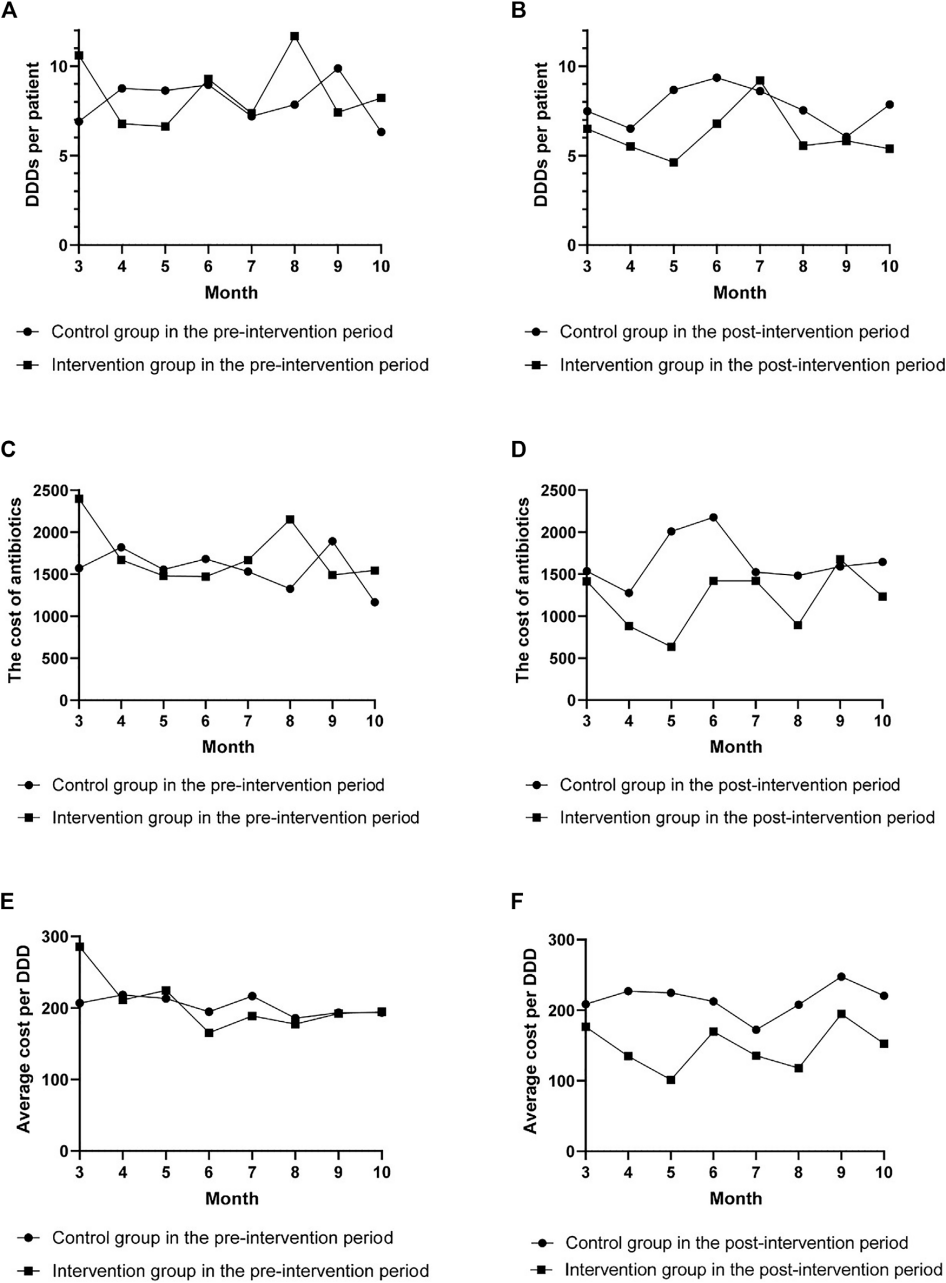
The change in defined daily doses (DDDs) per patient, cost of antibiotics, and average cost per DDD per month. The change in DDDs per patient **(A)**, cost of antibiotics **(C)**, and average cost per DDD **(E)** per month in the pre-intervention period. The change in DDDs per patient **(B)**, cost of antibiotics **(D)**, and average cost per DDD **(F)** per month in the post-intervention period.

As shown in Figure 1, there were no differences between the two groups in the trends of DDDs per patient, cost of antibiotics, and average cost per DDD during the pre-intervention period (*p* = 0.588, *p* = 0.285, and *p* = 0.876), while during the post-intervention period, significant differences in the trends between the two groups were observed (*p* = 0.025, *p* = 0.014, and *p* = 0.000, respectively, for DDDs per patient, cost of antibiotics, and average cost per DDD). The effects of the intervention on antibiotic consumption, costs, and length of hospital stay in the control and intervention groups between the pre-intervention and post-intervention period are shown in Tables 2 and 3. The DID results suggested that the intervention group was associated with a reduction in the average consumption of antibiotics [1.84 DDDs (95% CI, −3.433 to −0.337); *p* = 0.017], cost of antibiotics [598.19 RMB (95% CI, −1,018.558 to −173.554); *p* = 0.006], and cost per DDD [70.5 RMB (95% CI, −101.086 to −39.915); *p* = 0.000]. There were no significant differences in mean change of total costs and length of stay between the two groups.

**TABLE 2:** The mean antibiotic consumption, costs, and length of hospital stay in the control and intervention groups between the pre-intervention and post-intervention periods.

Outcomes	Pre-intervention		Post-intervention	
	Control group	Intervention group	Control group	Intervention group
DDDs per patient (DDDs)	7.93	8.29	7.67	6.19
The cost of antibiotics (RMB)	1546.99	1706.27	1624.51	1185.60
Total cost (RMB)	34529.55	27342.66	30792.38	30366.09
Average cost per DDD (RMB)	202.45	204.07	216.01	147.13
Length of stay (days)	10.85	11.30	10.92	12.38

**TABLE 3:** Difference-in-differences (DID) results of the net effects of antimicrobial stewardship (AMS) program on antibiotic consumption, costs, and length of hospital stay.

Outcomes	Coefficient	*p*-Value	95% CI	Robust SE
DDDs per patient	−1.885	0.017	(−3.433, 0.337)	0.789
The cost of antibiotics	−596.056	0.006	(−1018.558, −173.554)	215.305
Total cost	2320.662	0.476	(−4065.250, 8706.575)	3254.235
Average cost per DDD	−70.501	0.000	(−101.086, −39.915)	15.586
Length of stay	0.813	0.375	(−0.986, 2.611)	0.916

Note. DID, difference-in-differences.

### Construction of the Scoring System Involving Weights of Different Items

Table 4 presents the detailed results of the scoring system for perioperative antimicrobial prophylaxis, non-surgical antimicrobial prophylaxis, and therapeutic use of antibiotics. Among the items of the scoring system for perioperative antimicrobial prophylaxis and non-surgical antimicrobial prophylaxis, the weight of indication is the highest. On the other hand, timing of the initial dose and duration is assigned a lower weight. Regarding therapeutic use of antibiotics, the weight of indication is the highest, followed by choice, and dosage in its scoring system.

**TABLE 4:** The scoring system involving weights of different items.

Items	Perioperative antimicrobial prophylaxis	Non-surgical antimicrobial prophylaxis	Therapeutic use of antibiotics
Indication	0.41	0.57	0.37
Choice	0.16	0.21	0.23
Timing of the initial dose	0.07	NA	NA
Dosage and dosing interval	0.11	0.13	NA
Dosage	NA	NA	0.12
Dosing frequency	NA	NA	0.10
Duration	0.25	0.09	0.06
Intravenous-to-oral conversion	NA	NA	0.05
Combination of antibiotics	NA	NA	0.07

Note. NA, not available.

**FIGURE 2:**
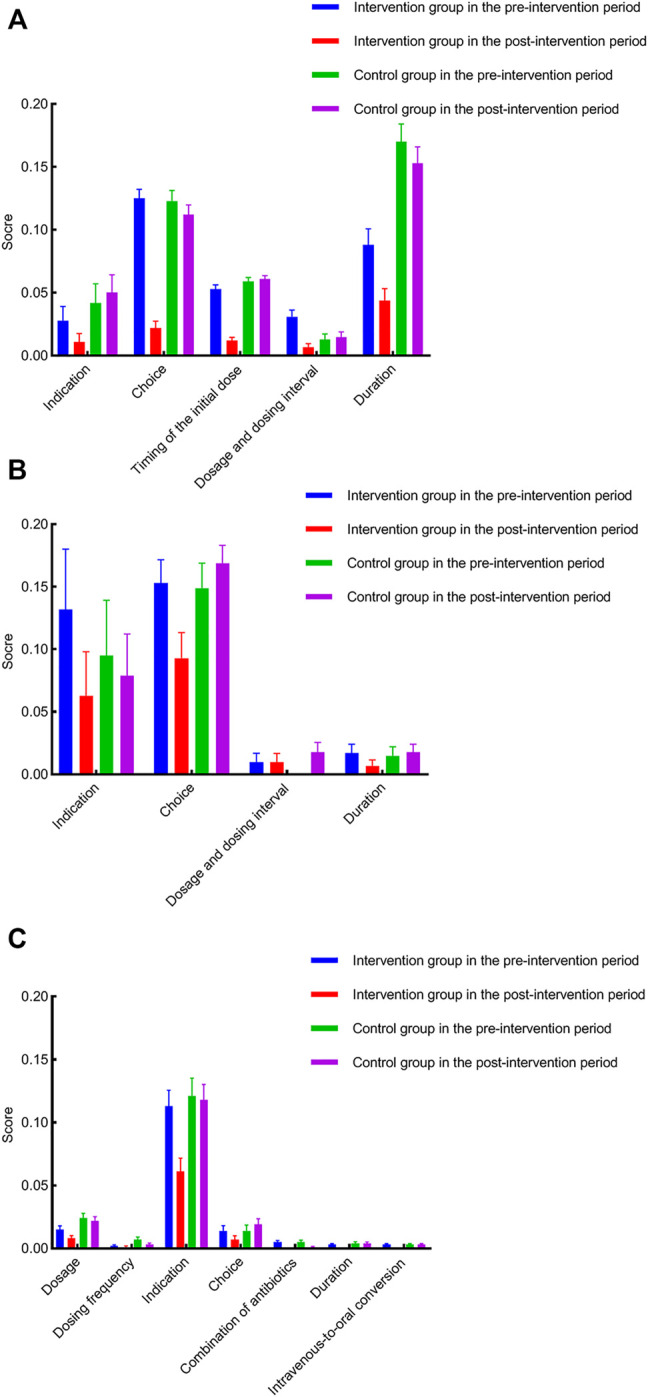
Category analysis for irrational use of antibacterial drugs. **(A)** inappropriate score of antimicrobial prophylaxis with respect to corresponding items for both groups during the pre- and post-intervention periods; **(B)** inappropriate score of non-surgical antimicrobial prophylaxis with respect to corresponding items for both groups during the pre- and post-intervention periods; **(C)** inappropriate score of antimicrobial treatment with respect to corresponding items for both groups during the pre- and post-intervention periods.

Figure 2 shows the detailed category analysis for irrational use of antibacterial drugs. The highest inappropriate score of items for the intervention group was antibiotic choice (0.12), antibiotic choice (0.15), and indication (0.11) in perioperative prophylaxis, non-surgical prophylaxis, and treatment during the pre-intervention period, respectively. After the intervention, inappropriate score of the corresponding items declined by 0.1, 0.06, and 0.05, respectively. Considering the antibiotic choice, as reported in Supplementary Table S1, the most frequently used antibiotics for perioperative prophylaxis in the intervention group before the intervention was cefathiamidine (37.80%). However, the use of cefathiamidine dropped to 1.90% during the post-intervention period. Meanwhile, cefazolin became the most frequently prescribed antibiotics, which increased from 2.44% to 50.48%. In terms of non-surgical prophylaxis, there was an increase in ceftriaxone use from 0 to 37.04% with a decrease in cefmetazole from 53.85% to 18.52% in the intervention group after the intervention.

### Evaluation of the Rationality of Antibiotic use

The inappropriate rate of antibiotic prescriptions, average inappropriate score for both groups, and associated parameters of DID analysis between the groups in perioperative antimicrobial prophylaxis, non-surgical antimicrobial prophylaxis, and therapeutic use of antibiotics during the pre-intervention and post-intervention period are shown in Tables 5 and 6. For the intervention group, inappropriate rate of antibiotic prescriptions in perioperative prophylaxis, non-surgical prophylaxis, and treatment decreased by 48.10%, 40.59%, and 29.25%, respectively. For the control group, inappropriate rate of antibiotic prescriptions in perioperative prophylaxis and non-surgical prophylaxis increased by 4.35% and 2.77%, respectively, while a 2.49% decrease in inappropriate rate of antibiotic prescriptions regarding treatment. During the post-intervention period, the average inappropriate score of perioperative antimicrobial prophylaxis in the intervention group declined by 0.23, while it decreased by 0.02 in the control group [0.21 (95% CI, −0.271 to −0.143); *p* = 0.000]. The average inappropriate score of non-surgical antimicrobial prophylaxis in the intervention group declined by 0.14, while it increased by 0.02 in the control group [0.16 (95% CI, −0.288 to −0.035); *p* = 0.010]. In terms of therapeutic use of antibiotics, the average inappropriate score of the intervention group declined by 0.07, while it decreased by 0.01 in the control group [0.06 (95% CI, −0.115 to −0.022); *p* = 0.003].

**TABLE 5:** Inappropriate rate of antibiotic prescriptions and average score for both groups during the pre-intervention and post-intervention periods.

Categories	Groups	Pre-intervention		Post-intervention	
		Inappropriate rate (%)	Average score	Inappropriate rate (%)	Average score
Perioperative antimicrobial prophylaxis^a^	Control group	95.65 (66/69)	0.41	100.00 (90/90)	0.39
	Intervention group	89.77 (79/88)	0.33	41.67 (45/108)	0.10
Non-surgical antimicrobial prophylaxis	Control group	91.67 (22/24)	0.26	94.44 (34/36)	0.28
	Intervention group	96.15 (25/26)	0.31	55.56 (15/27)	0.17
Therapeutic use of antibiotics	Control group	66.67 (100/150)	0.18	64.18 (129/201)	0.17
	Intervention group	57.06 (105/184)	0.15	27.81 (47/169)	0.08

Note. ^a^Antimicrobial use in some patients involving both prophylaxis and treatment.

**TABLE 6:** DID analysis of average score for both groups during the pre-intervention and post-intervention periods.

Outcomes	Coefficient	*p*-Value	95% CI	Robust SE
Perioperative antimicrobial prophylaxis	−0.207	0.000	(−0.271, −0.143)	0.033
Non-surgical antimicrobial prophylaxis	−0.164	0.010	(−0.288, −0.035)	0.040
Therapeutic use of antibiotics	−0.069	0.003	(−0.115, −0.022)	0.024

Note. DID, difference-in-differences.

### Pharmacists’ Recommendations Made in the Post-intervention Period

Over the 8-month post-implementation of a pharmacist-led AMS program, a total of 445 interventions were recommended, of which 321 (72.13%) were from medical order review led by clinical pharmacists (Table 7). Overall, 445 interventions were made with an average acceptance rate of 80.00% (356/445). The highest proportion of intervention types in perioperative antimicrobial prophylaxis was stopping due to prolonged duration (34.97%) with an acceptance rate of 66.67%. Among the recommendations for non-surgical prophylaxis, the most frequent type was initiating a preferred agent for indication, which had an acceptance rate of 47.62%. The most common intervention types for antibiotic treatment were discontinuation of antibiotics without indication (23.58%), followed by dose or frequency adjustment (19.10%), and de-escalation (13.82%). The acceptance rates were 67.24%, 91.49%, and 88.24%, respectively.

**TABLE 7:** Source, initiator, type, and acceptance rate of antimicrobial stewardship interventions.

Interventions	Perioperative antimicrobial prophylaxis	Non-surgical antimicrobial prophylaxis	Therapeutic use of antibiotics
Source of interventions	Number (%)		
Ward round consultation	18 (11.04%)	13 (36.11%)	54 (21.95%)
WeChat consultation	6 (3.68%)	2 (5.55%)	10 (4.07%)
Medical order review	139 (85.28%)	21 (58.33%)	161 (65.45%)
Multidisciplinary consultation	NA	NA	21 (8.54%)
Initiator of interventions	Number (%)		
Clinician	24 (14.72%)	15 (41.67%)	85 (34.55%)
Clinical pharmacist	139 (85.28%)	21 (58.33%)	161 (65.45%)
Type and acceptance of interventions	Accepted% (number accepted/number)		
Initiate preferred agent for indication	85.29% (29/34)	47.62% (10/21)	90.00% (9/10)
Dose/frequency adjustment	82.14% (23/28)	50.00% (2/4)	91.49% (43/47)
Drug timing adjustment	79.55% (35/44)	NA	NA
Stop due to prolonged duration	66.67% (38/57)	75.00% (3/4)	85.71% (6/7)
Discontinuation of antibiotics without indication	NA	57.14% (4/7)	67.24% (39/58)
Stop combination of antimicrobial treatment	NA	NA	87.50% (7/8)
Change antibiotic due to toxicity, allergy, etc	NA	NA	87.50% (7/8)
Escalation	NA	NA	100.00% (16/16)
De-escalation	NA	NA	88.24% (30/34)
Sequential intravenous-to-oral therapy	NA	NA	100% (11/11)
Imaging/laboratory/etiological examination	NA	NA	93.10% (27/29)
Therapeutic drug monitoring	NA	NA	94.44% (17/18)
Total	76.69% (125/163)	52.78% (19/36)	86.18% (212/246)

Note NA, not available.

## Discussion

To our knowledge, this is the first study to evaluate the impact of a pharmacist-led AMS program in the department of vascular and interventional radiology. The DID analysis indicated that the multifaceted pharmacist-led AMS intervention was associated with a significant decline in the antibiotic use, cost of antibiotics, and irrationality of antibiotic prescribing. Comparison of pre- and post-intervention changes between the intervention group and the control group, rather than merely levels before and after the intervention in the intervention group, necessarily adjusted for the possibility that the outcome was changing without the benefit of the intervention (Gerber et al., 2013).

There was a significant reduction in antibiotic utilization after the interventions in our study. This decline was attributed to reduction of unnecessary antimicrobial prescribing and increase in compliance with duration of antibiotic use. This observation was consistent with studies conducted in hospital and clinic settings (Hwang and Kim, 2018; Lee et al., 2018; Abubakar et al., 2019; Haseeb et al., 2020). The current study also found that the implementation of antibiotic stewardship interventions led to cost savings of antibiotics, which was similar to that found in prior studies (Butt et al., 2019; Xin et al., 2019). Some studies reported that the length of hospital stay significantly was shortened after the intervention of a pharmacist-directed antimicrobial stewardship (Mahmoudi et al., 2019; Xin et al., 2019; Arensman et al., 2020; Mahrous et al., 2020), while some studies indicated that the intervention had no effect on the length of stay (Wenzler et al., 2017; Abubakar et al., 2019; Monmaturapoj et al., 2021), which may be related to differences in the study design and analytical methods.

Inappropriate antibiotic prescribing was evaluated using a scoring system consisting of different items regarding three categories. Compared with the scoring system constructed by Shen et al. (2011), the relative weight values of each item were determined on the basis of utilizing the Saaty1–9 scale method, which can quantitatively judge the importance of each item on the inappropriate antibiotic prescribing. The result implies that indication had the largest impact for irrationality of antibiotics, regardless of antibiotic use for prophylaxis or treatment. Several studies showed that intervention has a significant impact on the appropriateness of antibiotic use after the implementation of the AMS program (Brink et al., 2017; Foolad et al., 2018; Butt et al., 2019; Calò et al., 2021; Jantarathaneewat et al., 2021). Our study was consistent with these findings, as we observed that the irrationality of antimicrobial prescriptions decreased in perioperative antimicrobial prophylaxis, non-surgical antimicrobial prophylaxis, and therapeutic use of antibiotics.

Inappropriateness of antimicrobial use in perioperative prophylaxis significantly decreased, especially in first-choice antibiotics, which was similar to that found in a previous study (Yang et al., 2017). Regarding the choice of antimicrobial prophylaxis, cefazolin definitely became the first choice in the post-intervention period with a concomitant decrease in cefathiamidine. Indeed, cefazolin was recommended as the first-line antibiotic according to the evidence-based trials (National Health Commission of China, 2015). Previous studies have reported that implementation of antibiotic stewardship program in surgeries was associated with reduction in the prescription of broad-spectrum and expensive antibiotics (Dona et al., 2019; Mahmoudi et al., 2019).

Inappropriateness score of antimicrobial prescribing for non-surgical prophylaxis decreased, manifested by improvement in correct antibiotic choice. The current study observed an increased use of ceftriaxone instead of cefoxitin for cirrhotic patients with esophageal and gastric variceal bleeding. Cirrhotic patients presenting with acute variceal bleeding have a high risk of developing bacterial infections; therefore, initiation of prophylactic antibiotic treatment at the time of admission is necessary (Lee et al., 2014). According to the guidelines (Korean Association for the Study of the, 2020), short-term antibiotic prophylaxis with intravenous ceftriaxone is recommended in cirrhotic patients with variceal bleeding.

A significant reduction in inappropriate prescribing for therapeutic use of antibiotics was also found after the intervention, manifested by a reduction in inappropriate prescribing in conditions for which antibiotics were generally not indicated, such as superficial thrombophlebitis of the lower extremities. Other antimicrobial stewardship efforts also have demonstrated improvements in unnecessary antibiotic prescribing for viral acute respiratory infection and asymptomatic bacteriuria (Meeker et al., 2016; Akpan et al., 2020; Calò et al., 2021; May et al., 2021). Antibiotic prescribing for these conditions where antimicrobials are not indicated puts them at unnecessary risk for adverse events and should be a target for quality improvement in interventional radiology wards.

In the intervention period, the acceptance rate of interventions was 80.00%, which was similar to that described in other studies at hospitals (Li et al., 2017; Hurst et al., 2019). The highest proportion of intervention types for surgical prophylaxis during the intervention period was stopping antimicrobial treatment, but the acceptance rate was the lowest. The reason is as follows: first, according to the Clinical Practice Guidelines for antibiotic prophylaxis during vascular and interventional radiology procedures (Chehab et al., 2018), a single preprocedural dose of cefazolin is an effective regimen for arterial endografts in the management of aortic aneurysm or dissection. Chinese Guidelines also suggest a single dose for prophylaxis (National Health Commission of China, 2015). However, the aortic stent graft surgery involves important viscera, an endograft infection that carries high morbidity and mortality rates (as high as 27%) (Cernohorsky et al., 2011). Therefore, doctors are unwilling to use a single dose of antibiotics. Second, postoperative inflammatory response syndrome may occur after aortic stent graft procedures and hepatic artery chemoembolization, which is characterized by fever, leukocytosis, and elevated C-reactive protein (CRP) and other inflammatory markers (Blackburn and West, 2016; Daye and Walker, 2018). Sometimes it is difficult for doctors to distinguish it from infection, so they dare not easily stop the antibiotics. Compared with perioperative antimicrobial prophylaxis, non-surgical antimicrobial prophylaxis had a lower acceptance rate for intervention, which was mainly manifested in the recommendation of using preferred evidence-based drug. That is probably because clinicians in the surgical ward might pay more attention to perioperative antimicrobial prophylaxis that resulted in change of prescribing behavior. In terms of therapeutic use of antibiotics, the lowest acceptance rate of intervention type was discontinuation of therapy without indication. For example, superficial thrombophlebitis is characterized by the appearance of tenderness, pain, redness, and swelling in the affected veins, which refers to an inflammation of superficial veins. Nonsteroidal anti-inflammatory drugs are suggested to reduce inflammation rather than antibiotics (Di Nisio et al., 2018). However, the strained relationship between doctors and patients in China has made doctors hesitant to not use antibiotics.

Our study has several strengths. Although the AMS program was performed in a surgical ward, a comprehensive intervention implemented not only focused on perioperative antimicrobial prophylaxis but also on non-surgical antimicrobial prophylaxis and therapeutic use of antibiotics. Notably, a scoring system was developed for assessment of rationality of antimicrobial prescriptions in antimicrobial prophylaxis and treatment based on consultation of multidisciplinary experts experienced in the management of infectious diseases. Previous studies usually emphasized on the analysis of change trend in antimicrobial utilization and cost savings after the implementation of AMS, whereas a comprehensive evaluation of antimicrobial prescriptive appropriateness was conducted as well as antimicrobial consumption and cost in our study.

There were also some limitations. First, it was a nonrandomized, retrospective pre–post analysis that carry a risk of selection bias. However, a quasi-experimental design with a DID methodology is often used to estimate the net effect of the intervention. Second, the present study investigated only the short-term effects of a pharmacist-led AMS intervention in the department of vascular and interventional radiology. It is unclear whether these effects would sustain after the interventions had finished. Third, this study was limited by its sample size. Finally, this was a single center study conducted in a tertiary teaching hospital; hence, the possibility of generalizing our results to other hospitals was limited. Future study with a larger sample size and a more rigorous design over an extended period needs to be confirmed. In spite of the limitations, this study has demonstrated that implementation of pharmacist-led AMS program significantly decreased the antibiotic use, cost of antibiotics, and irrationality of antibiotic prescribing in the department of vascular and interventional radiology.

## Conclusion

This study provides important evidence from a pre-post intervention study that implementation of AMS interventions was associated with a marked reduction of antibiotic use, cost of antibiotics, and irrationality of antibiotic prescribing in the department of vascular and interventional radiology in a Chinese hospital. Our data indicate that departments that are not a priority for antimicrobial administration also need attention. It is essential for surgeons to be aware of the importance of adherence to guidelines. The findings of our study indicate that the beneficial AMS intervention led by clinical pharmacists can help to promote the rational use of antibiotics and decrease the antibiotic utilization.

## Data Availability

The original contributions presented in the study are included in the article/Supplementary Material, further inquiries can be directed to the corresponding authors.
