# Supplementary material for: The Impact of a Multifaceted Pharmacist-Led Antimicrobial Stewardship Program on Antibiotic Use: Evidence From a Quasi-Experimental Study in the Department of Vascular and Interventional Radiology in a Chinese Tertiary Hospital
Source: Front Pharmacol. 2022 Feb 28;13:832078. doi: 10.3389/fphar.2022.832078 (PMC8919369; doi:10.3389/fphar.2022.832078)
Supplement: Supplementary file 1 [file Table1.DOCX]

Supplementary Table S1. Comparison of patterns of antimicrobial prescriptions in the pre- and post-intervention phases

| Antibiotic | Perioperative antimicrobial prophylaxis | | | | Non-surgical antimicrobial prophylaxis | | | | Antibiotics for therapeutic use of antibiotics | | | |
| --- | --- | --- | --- | --- | --- | --- | --- | --- | --- | --- | --- | --- |
|  | Control group | | Intervention group | | Control group | | Intervention group | | Control group | | Intervention group | |
|  | Pre-intervention | Post-intervention | Pre-intervention | Post-  intervention | Pre-intervention | Post-intervention | Pre-intervention | Post-  intervention | Pre-intervention | Post-intervention | Pre-intervention | Post-  intervention |
| Amoxicillin-clavulanic  Acid (I.V)^a^ | NA | NA | NA | 7(6.67%) | NA | NA | NA | NA | 3(1.68%) | 2(0.87%) | 15(5.19%) | 14(5.43%) |
| Amoxicillin(P.O)^b^ | NA | NA | NA | NA | NA | NA | NA | NA | NA | NA | NA | 1(0.39%) |
| Penicillin(I.V) | NA | NA | NA | NA | NA | NA | NA | NA | 1(0.56%) | 5(2.18%) | 1(0.35%) | 1(0.39%) |
| Cefazolin(I.V) | NA | NA | 2(2.44%) | 53(50.48%) | NA | NA | NA | NA | NA | NA | NA | 1(0.39%) |
| Cefathiamidine(I.V) | 1(1.61%) | NA | 31(37.80%) | 2(1.90%) | NA | NA | NA | NA | 3(1.68%) | NA | 11(3.81%) | NA |
| Cefprozil(P.O) | NA | NA | NA | NA | NA | NA | NA | NA | 1(0.56%) | NA | 10(3.46%) | 21(8.14%) |
| Cefuroxime(I.V) | NA | 1(1.23%) | NA | 3(2.86%) | NA | NA | NA | NA | 2(1.12%) | 3(1.31%) | 3(1.04%) | 8(3.10%) |
| Cefuroxime(P.O) | NA | NA | NA | NA | NA | NA | NA | NA | NA | NA | 1(0.35%) | NA |
| Cefaclor(P.O) | NA | NA | NA | NA | NA | NA | NA | NA | NA | NA | NA | 2(0.78%) |
| Cefixime(P.O) | NA | NA | NA | NA | NA | NA | NA | NA | NA | NA | NA | 6(2.32%) |
| Ceftizoxime(I.V) | 11(17.74%) | 10(12.35%) | 27(32.93%) | 2(1.90%) | 7(29.17%) | 4(11.11%) | NA | NA | 33(18.54%) | 26(11.35%) | 77(26.64%) | 17(6.59%) |
| Ceftriaxone((I.V) | NA | NA | NA | NA | NA | NA | NA | 10(37.04%) | 1(0.56%) | NA | 1(0.35%) | NA |
| Cefmetazole(I.V) | 29(46.77%) | 33(40.74%) | 14(17.07%) | 6(5.71%) | 7(29.17%) | 12(33.33%) | 14(53.85%) | 5(18.52%) | 85(47.75%) | 91(39.74%) | 42(14.53%) | 39(15.12%) |
| Cefoxitin(I.V) | 21(33.87%) | 37(45.68%) | 8(9.76%) | 31(29.52%) | 10(41.67) | 20(55.56%) | 12(46.15%) | 12(44.44%) | 30(16.85%) | 70(30.57%) | 42(14.53%) | 74(28.68%) |
| Piperacillin-tazobactam(I.V) | NA | NA | NA | NA | NA | NA | NA | NA | NA | 2(0.87%) | 3(1.04%) | 11(4.26%) |
| Cefoperazone-sulbactam(I.V) | NA | NA | NA | NA | NA | NA | NA | NA | NA | 2(0.87%) | 2(0.69%) | 8(3.10%) |
| Moxifloxacin(I.V) | NA | NA | NA | NA | NA | NA | NA | NA | 6(3.37%) | 5(2.18%) | 12(4.15%) | 3(1.16%) |
| Moxifloxacin(P.O) | NA | NA | NA | NA | NA | NA | NA | NA | NA | 1(0.44%) | NA | 6(2.32%) |
| Levofloxacin(I.V) | NA | NA | NA | NA | NA | NA | NA | NA | NA | 3(1.31%) | NA | 12(4.65%) |
| Meropenem(I.V) | NA | NA | NA | NA | NA | NA | NA | NA | 2(1.12%) | 1(0.44%) | 3(1.04%) | 14(5.43%) |
| Imipenem-cilastatin(I.V) | NA | NA | NA | NA | NA | NA | NA | NA | 3(1.68%) | 1(0.44%) | 4(1.38%) | 2(0.78%) |
| Biapenem(I.V) | NA | NA | NA | NA | NA | NA | NA | NA | 3(1.68%) | 7(3.06%) | 30(10.38%) | 10(3.88%) |
| Vancomycin(I.V) | NA | NA | NA | NA | NA | NA | NA | NA | 2(1.12%) | 6(2.62%) | 4(1.38%) | 5(1.94%) |
| Teicoplanin(I.V) | NA | NA | NA | NA | NA | NA | NA | NA | NA | 1(0.44%) | 2(0.69%) | 1(0.39%) |
| Linezolid(I.V) | NA | NA | NA | NA | NA | NA | NA | NA | NA | 1(0.44%) | NA | NA |
| Metronidazole(I.V) | NA | NA | NA | NA | NA | NA | NA | NA | NA | NA | 2(0.69%) | 1(0.39%) |
| Ornidazole(I.V) | NA | NA | NA | NA | NA | NA | NA | NA | 2(1.12%) | 1(0.44%) | 21(7.27%) | NA |
| Azithromycin(I.V) | NA | NA | NA | NA | NA | NA | NA | NA | NA | 1(0.44%) | NA | 1(0.39%) |
| Clindamycin(I.V) | NA | NA | NA | 1(0.95%) | NA | NA | NA | NA | NA | NA | 1(0.35%) | NA |
| Etimicin(I.V) | NA | NA | NA | NA | NA | NA | NA | NA | 1(0.56%) | NA | 2(0.69%) | NA |
| gentamycin(I.V) | NA | NA | NA | 1(0.95%) | NA | NA | NA | NA | NA | NA | NA | NA |

NA, not available.

^a^Intravascular (I.V)

^b^Per Oral (P.O)
